# Prevalence, Fate and Effects of Plastic in Freshwater Environments: New Findings and Next Steps

**DOI:** 10.3390/toxics8030072

**Published:** 2020-09-17

**Authors:** Farhan R. Khan

**Affiliations:** Department of Science and Environment, Roskilde University, Universitetsvej 1, PO Box 260, DK-4000 Roskilde, Denmark; frkhan@ruc.dk or farhan.khan@gmx.com; Tel.: +46-5019-0429

At a time when a global pandemic rightly holds our collective attention, environmental issues have taken a backseat to the ongoing battle against Covid-19. However, when normality resumes (or a new normal emerges), these environmental concerns will return to the fore and few issues are as tangible or emotive as plastic pollution. The presence of plastics in aquatic environments, particularly freshwater environments that are relied upon on a daily basis (e.g., for water and food supply, as well as recreation and tourism), is a constant reminder of the indelible mark that our species is leaving on the planet. Indeed, whilst plastic pollution has been described as a potential hallmark of the Anthropocene [1], our understanding of its presence and impacts on freshwater systems still lags far behind the corresponding knowledge that has been amassed from marine (coastal and oceanic) locales [2,3,4]. In this Special Issue on the “Prevalence, Fate and Effects of Plastic in Freshwater Environments”, original research articles report the latest findings describing the occurrence of plastics and microplastics (MPs, <5 mm in size) within previously uninvestigated rivers and lakes. As important as mapping plastics pollution across global freshwaters is gaining an understanding into the effects of the interactions between MPs, formed from the degradation of larger debris (secondary MPs) or purposely manufactured microscopic particles (primary MPs), and biota. Laboratory-based mechanistic studies examined the effects of MP exposure to freshwater organisms, with endpoints ranging from cellular to behavioral. This Editorial aims to highlight the papers presented within the Special Issue and place them into the broader context of freshwater plastics research.

To date, few studies reporting plastic and MP prevalence in freshwaters have originated from the African continent [4], despite the presence of some of the world’s most notable lakes and rivers. This knowledge gap needs to be urgently addressed [5] and thus it was especially pleasing that research from Lake Malawi and the River Nile contributed to this Special Issue. Focusing on macro-sized anthropogenic litter, the study by Mayoma et al. [6] resulted from an NGO-led citizen science project to clean up the shorelines of Lake Malawi. Over 2000 volunteers participated in four annual beach clean-ups from 2015 to 2018. Almost half a million individual items of beach debris were collected, of which approximately 80% was plastic. Glass, metal and paper were also found at each site, but plastic litter was by far the most abundant. The composition of plastic litter varied between sites, but carrier bags (22.5–49.4%), personal hygiene products (7.15–18.6%), beverage bottles (1.9–18.8%) and discarded fishing gear (0.21–20.25%) represented the major categories. The study highlighted the important role that volunteers could play in the remediation of the environment and the collection of scientific data, as has been discussed elsewhere for freshwater systems [7]. Moreover, by focusing on macroplastics in the African Great Lakes, the study provides a link between the use (misuse) of plastic products on the shoreline and the ingestion of MPs by resident lake species, as has been noted elsewhere in the region (Lake Victoria, [8]).

The research conducted in Lake Victoria was the first to describe the ingestion of MPs by African freshwater species (Nile perch and Nile tilipia) [8] and, although subsequent similar research from different parts of the continent has emerged, documenting the occurrence of MPs in the Nile River, the world’s longest river, and its biota has been a surprising knowledge gap. The Nile River flows from the heart of Africa to the Mediterranean Sea, but the presence of MPs was determined in Cairo, Egypt’s capital, with an estimated population of 20 million people. Analyzing the digestive tracts of Nile tilapia (*Oreochromis niloticus*) and catfish (*Bagrus bayad*) revealed the presence of MPs in over 75% of the sampled fish [9]. Similar studies from both marine and freshwater environments showed that this high level of MP ingestion is rarely found and that fish sampled from the Nile River in Cairo are potentially among the most in danger of consuming MPs worldwide. The gastrointestinal tracts of omnivorous tilipia contained significantly more MPs than those of the piscivorous catfish, which is likely due to the former’s more varied diet and increased potential to mistake plastic items for food. In keeping with similar studies, fibers were the most abundant MP type, followed by films and fragments. The diverse origins of fibers that may result from the degradation of clothing, furniture or fishing gear means that it is difficult to pinpoint any one source of MPs beyond general urbanization. Polyethylene (PE), polyethylene terephthalate (PET) and polypropylene (PP) were all identified, and these polymers are widely used in a plethora of plastic products. The first detailing of MPs in fish from the Nile River revealed the scale of the problem, but further research is needed understand and mitigate its effects.

As famous as the Nile River is the Mississippi River (USA). During periods of flooding along the lower Mississippi River, its waters are diverted through the Bonnet Carré Spillway into the coastal area of the Mississippi Sound, which prevents flooding downstream in the city of New Orleans. Such a major flooding event occurred during 2019. The study by Scircle et al. (2020) [10] captured the effect of this on the presence of MPs in the waters housing oyster (*Crassostrea virginica*) reefs in the Mississippi Sound. Although coastal, during the spring and summer of the 12 month sampling campaign, historic flooding of the Mississippi River caused major freshwater intrusions into the sampled locations. Across the ten sample sites and seasonal sampling periods, there was considerable spatial and temporal variation, as might be expected, with the average MP concentration ranging from 30 to 192 MPs/L across sites. During the period of freshwater intrusions, MP abundances tended to correlate with salinity. In other words, sites most impacted by freshwater intrusions had the least MPs owing to a dilution effect, but this effect was temporary. There were no significant changes in the relative distribution of MPs during freshwater intrusions, with most of the MPs (>50%) in the lower size fraction (~25–90 µm). Fragments (~84%), fibers (~11%) and beads (~5%) constituted the MP types. Polyester, acrylates/polyurethanes, polyamide, polypropylene, polyethylene and polyacetal were all identified polymers. This work provides empirical data on the waters of the Mississippi Sound, in which oyster reefs are located. More research is needed to determine the impacts on the organisms. However, this investigation importantly demonstrates how MP pollution is impacted by changing conditions and thus represents a counterpoint to the numerous studies that only capture MP pollution during a ‘single snapshot in time’. Temporal and seasonal events need greater recognition.

The study from the Nile River [9] illustrates, again, if further proof was necessary, the already established phenomena of fish indiscriminately ingesting MPs, but the impacts on the fish are not fully understood. Inflammatory responses and intestinal health by way of alterations of the microbiome resulting from MP exposure constitute one such area that requires further elucidation. In the study by Kurchaba et al. (2020) [11], larval zebrafish (5 days post fertilization) were exposed to PE MPS for four or ten days. Markers of metabolic disturbance and inflammation were assessed through physiological and genomic analysis. The ten-day exposure to 20 mg MPs/L did not seem to affect the overall metabolism of the larval zebrafish and had limited impact on inflammatory molecular responses. However, it appeared to trigger an elevated reactive oxygen species (ROS) response, with a significant increase in the oxidative stress mediator L-FABP (liver fatty-acid binding protein). This increase in ROS was accompanied by a higher abundance of *Bacteriodetes*, and although further research is needed to verify the link between these two outcomes, the authors speculated that increased oxidative stress in MP fish promotes the growth of these bacteria. Localization of ROS and concurrent dysbiosis of the larval microbiome indicate that aquatic organisms are negatively affected by MP exposure, which may render the animal more susceptible to diseases. The gut microbiome has also emerged as a new growth area in explaining adverse human health and disease. The finding that MP exposure disrupts the microbiome of zebrafish may have important ramifications for humans, especially since it is known that MPs have been found in a variety of foodstuffs.

The potential for oxidative damage following PE MP exposure was also assessed by Scopetani et al. (2020) [12] using the freshwater oligochaete *Tubifex tubifex*. Worms were exposed via water or sediment separately or simultaneously spiked with MPs. Mortality was assessed over 120 h and oxidative stress status was assessed via enzymatic assays for the biomarkers glutathione reductase and peroxidase. At concentrations used (2 g/L (*w/v*) in water and 2 mg/g (*w/w*) in sediment), *Tubifex* survival was not significantly affected. Similarly, there was no effect on the activities of either biomarker. Other species have been affected by MPs at similar concentrations and thus the results described in this study may be due to tolerance of the chosen test species. Importantly, though, the lack of effects should not diminish the findings and, as in the case of all new pollutants (including MPs), it is necessary that negative results are also made available so that associated risks may be discussed with nuance.

The use of biomarkers has been widely debated in ecotoxicology, with one area of concern being the link between intracellular responses and physiological or behavioral response. In the study by Pflugmacher et al. (2020) [13], the annelid *Enchytraeus crypticus* was subject to a choice experiment in which different amounts of high-density PE MPs taken from green bottle caps were mixed into soil. In each test, worms chose soil without MPs or the area with a lower MP concentration. Contact with MPs resulted in enhanced oxidative stress measured through the antioxidative enzymes catalase and glutathione S-transferase. Explaining the results, the authors noted that the exposure time was too short for chemicals to leach from the plastics and that the MPs were too large to be ingested. Thus, the likely cause of avoidance behavior was that the addition of MPs adversely changed the properties of the soil.

In summary, this collection of original research articles provides a valuable update of recent investigations into the global reach of plastic pollution within freshwaters and the biological effects induced by its presence. The question of how freshwater plastics research should proceed is of paramount importance, but there are no easy ‘next steps’. Clearly, more information is needed on the pervasive presence and effects of plastics in freshwaters, pursuing monitoring studies that include temporal and spatial factors that affect, for instance, hydrology and water flow and also accounting for a wider range of localized and systemic endpoints. As the studies within this Special Issues illustrate, there is broad range of topics to cover, and whilst calls have been made to harmonize and standardize plastics research, this belies the realities of working in such a diverse field and particularly the challenges in conducting scientific inquiry in different parts of the world. Approaches taken within each study must therefore endeavor to match the research aims of that singular investigation whilst simultaneously moving the research area towards a more robust and dynamic framework that can meet the future challenges of an ever-expanding group of anthropogenic particulates, including, but not limited to, the emergent concerns surrounding nanoplastics [14] and tire wear particles [15]. Moreover, returning to the opening remarks of this Editorial on the current pandemic, the outbreak of Covid-19 has increased the use and discard of personal protective equipment, which heightens the need to focus on plastic waste generation, its impacts and mitigation [16,17]; from its ecological and ecotoxicological impacts through to defining improved strategies for waste management. The studies presented in this Special Issue add to the weight of evidence that recognizes plastic pollution as a significant freshwater problem that requires immediate attention. The consequences of inaction are severe.
